# Supplementary material for: Splice-Junction-Based Mapping of Alternative Isoforms in the Human Proteome
Source: Cell Rep. Author manuscript; Available in PMC 2020 Jan 15. (PMC6961840; doi:10.1016/j.celrep.2019.11.026)
Supplement: 2 [file NIHMS1546469-supplement-2.zip › DF1/PXD006675/TricuspidValve/TricuspidValve_4_DPP7_GAHLLLHWER.pdf]

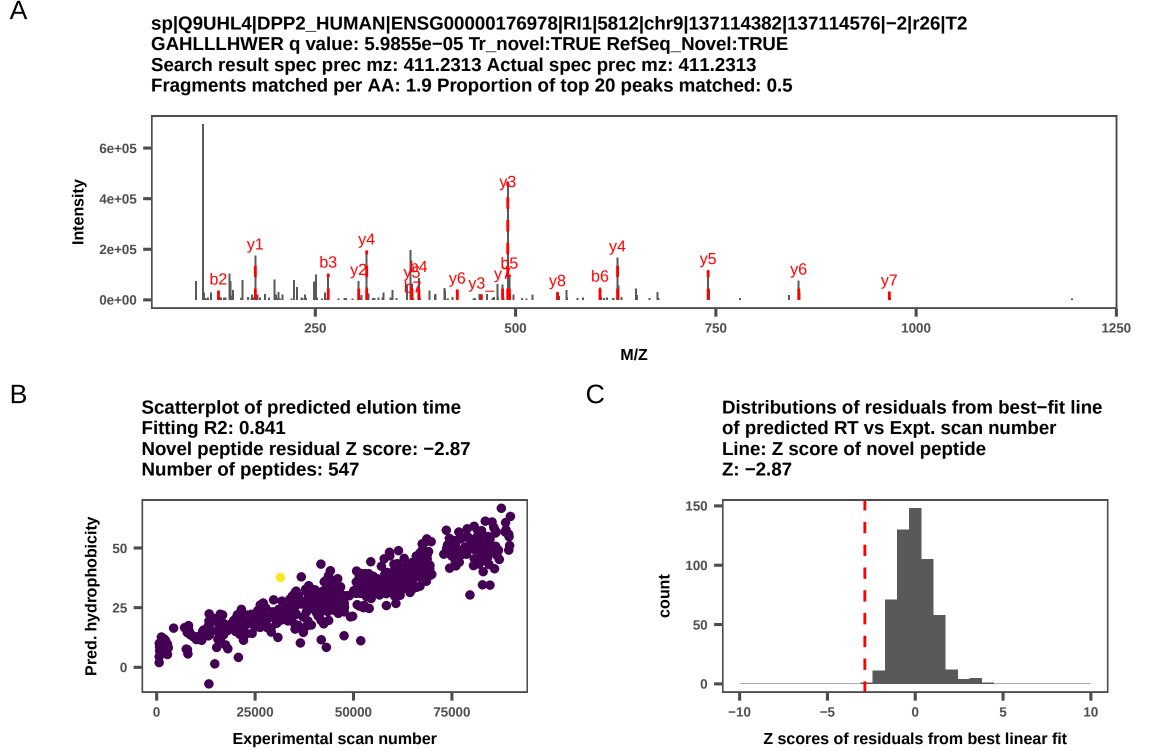
A
sp|Q9UHL4|DPP2_HUMAN|ENSG00000176978|RI1|5812|chr9|137114382|137114576|−2|r26|T2
GAHLLLHWER q value: 5.9855e−05 Tr_novel:TRUE RefSeq_Novel:TRUE
Search result spec prec mz: 411.2313 Actual spec prec mz: 411.2313
Fragments matched per AA: 1.9 Proportion of top 20 peaks matched: 0.5
Intensity
6e+05
4e+05
2e+05
0e+00
y3
y1
y4
b3
y2
b4
y4
y5
y6
b5
y6
b2
y8
b6
y7
250
500
750
1000
1250
M/Z
B
Scatterplot of predicted elution time
Fitting R2: 0.841
Novel peptide residual Z score: −2.87
Number of peptides: 547
Pred. hydrophobicity
50
25
0
0
25000
50000
75000
Experimental scan number
C
Distributions of residuals from best−fit line
of predicted RT vs Expt. scan number
Line: Z score of novel peptide
Z: −2.87
count
150
100
50
0
−10
−5
0
5
10
Z scores of residuals from best linear fit
